# Physical Activity, Taste Preferences, Selected Socioeconomic Characteristics: Differentiators of Consumer Behavior Among Older Adults in the Dairy Market in Poland—A Pilot Study

**DOI:** 10.3390/nu17071127

**Published:** 2025-03-24

**Authors:** Robert Gajda, Marzena Jeżewska-Zychowicz, Rafał Kubacki

**Affiliations:** 1 Department of Human Nutrition, Faculty of Biotechnology and Food Sciences, Wrocław University of Environmental and Life Sciences, Chełmońskiego 37, 51-630 Wroclaw, Poland; 2 Department of Food and Consumption Market Research, Faculty of Human Nutrition, Warsaw University of Life Sciences, Nowoursynowska 166, 02-787 Warsaw, Poland; marzena_jezewska_zychowicz@sggw.edu.pl; 3 Faculty of Physical Education and Sports, Wroclaw University of Health and Sport Sciences, Paderewskiego 35, 51-612 Wroclaw, Poland; rafal.kubacki@awf.wroc.pl

**Keywords:** consumer behavior, taste preferences, socioeconomic characteristics, physical activity, older adults

## Abstract

Background: Dairy products contain many nutrients that are important for the human body, as they serve to maintain its physiological functions and protect against many diseases. Their consumption by older adults, however, raises certain doubts, including the risks of lactase deficiency, milk protein allergy, etc. Functional dairy products can help maintain or increase the consumption of dairy products among the elderly. This study aimed to evaluate the relationship between older adults’ habitual purchases of dairy products and their taste preferences and frequency of buying functional dairy products, physical activity, and selected socioeconomic characteristics. Materials and methods: This study was conducted between July and October 2024, among 310 people aged 60 and over in Poland. The study collected data on the frequency of buying dairy products (the PF-DP scale), preferences (the P_DP scale), physical activity (the IPAQ questionnaire), and socio-demographic and economic characteristics. The PCA identified three patterns of buying behavior. The relationship between the identified buying behavior patterns and their determinants was verified using the Kruskal–Wallis test and Chi-square. Results: It was found that high intensity of the “conventional dairy products and fats” pattern correlated with high taste preferences (Me = 8.0; *p* < 0.05), living with family regardless of whether with or without a partner (11.8% and 15.8%; *p* = 0.002), high physical activity (MET = 5975.5; *p* = 0.004), including movement (MET = 1803.0; *p* = 0.028), sports and recreational activities (MET = 1908.0, *p* = 0.017), and frequent purchases of different functional food groups. The financial situation described as “we have an average standard of living” was related to the high intensity of the “dairy fat” pattern (62.3%; *p* = 0.018) and its moderate intensity to the high activity associated with movement (MET = 1788.0; *p* = 0.004). More than half of the sample never purchased functional dairy products. A high intensity of the “conventional dairy products and fats” pattern was associated with more frequent purchases of functional products compared to other patterns. Conclusions: The higher physical activity of older people was accompanied by a higher intensity of the “conventional dairy products and fats” pattern and more frequent purchases of functional dairy products. An evaluation of the relationships between the determinants and buying behaviors of older people in the dairy market, especially their causal nature, requires further research.

## 1. Introduction

Aging is an irreversible process associated with a decline in tissue and cell functions, as well as an increased risk of various age-related diseases [1,2,3,4], including musculoskeletal diseases that reduce physical fitness. The muscle mass of older people is influenced by, among others, environmental factors, factors related to physical exertion, and nutritional factors [5].

Milk and dairy products contribute to the improvement of the overall diet quality [6]. Dairy products are a nutrient-rich source of protein, the deficiency of which adversely affects muscle mass and strength [7]. Dairy provides several other nutrients that potentially protect against frailty, including calcium, magnesium, and vitamin D [8,9,10]. In addition, milk and its products are a source of magnesium, zinc, copper, molybdenum, and vitamins A, E, B_1_, B_2_, B_6_, B_12_, niacin, pantothenic acid, and folate [11], which are essential in the prevention and treatment of diseases associated with changes in body composition [2,3,12,13,14]. Research shows that the consumption of dairy products can help stave off movement restrictions [15,16]. The daily consumption of milk and milk products was inversely associated with functional disability in older men [17] and better women’s physical performance compared to non-consumers [18]. The epidemiological evidence is still inconsistent regarding the relationship between intakes of dairy products and frailty risk in older adults [19,20]. Dairy products positively affect frailty and sarcopenia [21,22]. However, milk and dairy product consumption has some limitations, related to lactose intolerance and allergy to milk components [23,24]. The atherogenic properties of dairy fat remain debatable due to inconclusive research results [24,25].

Many dairy food add a substantial amount of saturated fat to the diet, which have adverse effects on blood lipids and are positively associated with the risk of cardiovascular disease [26]. Other studies suggest that the consumption of dairy products by the elderly at recommended levels does not impair the body’s lipid metabolism [27]. Functional dairy products, which aim to reduce the adverse effects of consuming dairy products or increase their positive impact, are being introduced to the market [28,29] and are becoming more widely consumed by older adults [30]. Despite some controversy, the dietary guidelines in Poland and other countries recommend the daily consumption of dairy products as part of a healthy diet for the general population.

Many factors condition the consumption of milk and dairy products, including both macroeconomic factors [31,32,33] and microeconomic factors, i.e., socio-demographic and economic conditions of the consumer [34,35], as well as taste preferences [36,37,38]. Milk and its products have been a particularly valuable food group in the average Polish diet for centuries [39]. In studies conducted in Poland, particular attention was paid to the socioeconomic structure of households, family structure, income, education level, province, region, place of residence [34,35], and sensory preferences [34,36,37,38]. The socioeconomic status of older people is one of the most critical factors affecting not only the purchase of dairy products but also the intensity level of their consumption. This is primarily related to older people’s financial resources or household income and the possibility of receiving social support. The family structure is the second important factor significantly stimulating or limiting dairy product consumption [35]. The decisive impact of these characteristics of older adult’s households on the variation in the consumption of different groups of dairy products has been confirmed by a study of the Polish population [35,38].

Research results suggest that regular physical activity provides extensive health benefits in older adults [40]. Older adults, however, are often less physically active [41]. Only 2.5–22% of community-dwelling older adults achieve current WHO-recommended PA levels (150 min moderate-intensity PA per week) [42]. Barriers to older adults’ engagement with PA include pain and discomfort, concerns with falling, and access difficulties [43]. The consumption of dairy products can help stave off movement restrictions [15,16], although the evidence supporting this relationship is incomplete.

Incorporating milk and dairy products into an older person’s diet is practical and convenient. Most dairy products’ texture eliminates consumption barriers, such as chewing and swallowing problems. Their nutrient-rich nature, providing high-quality protein, micronutrients, and bioactive compounds, can promote a good quality of life, including physical activity. Since their consumption is conditioned by the socio-demographic and economic characteristics of the individual, these factors were taken into account, along with taste preferences, to explain the link between the purchase of milk and dairy products, as well as milk fats and physical activity in older people. This study evaluated the relationship between older people’s habitual purchase of dairy products and their taste preference, frequency of buying functional dairy products, physical activity, and selected socioeconomic characteristics.

## 2. Materials and Methods

### 2.1. Study Design and Sample

This cross-sectional pilot study was conducted between July and October 2024. All stages of the study involved 310 people aged 60 and over from the Lower Silesian province. Senior Organizations in Wroclaw, i.e., “Space of the Third Age” and “ALTIUS” Association, as well as the City and Municipality Offices in Siechnice and Święta Katarzyna, were asked to help recruit respondents. The study was conducted in three stages.

The first stage of the study included a “panel discussion” on understanding the questions. It was conducted in July 2024 among 30 people aged 60 and over at a selected Senior Club in Wroclaw. This stage of the study allowed changes to be made to the content of the questions and measurement scales in line with the comments of potential respondents.

Pretesting was the second stage of the study, and it was designed to assess the relevance and reliability of the test. This stage was carried out in July 2024 in 30 people aged 60 and over at a selected Senior Club in Wroclaw. The questionnaire was completed by respondents 2 weeks apart (test–retest). Pre-testing was conducted for two scales: PF_DP (Purchasing Frequency, Dairy Products) and P_DP (Preferences, Dairy Products). To verify the measurement accuracy of the tested variables, a reliability analysis was performed using Cronbach’s alpha method [44]. Cronbach’s alpha reliability coefficient for the PF_DP scale was estimated at α = 0.89 and for the P_DP scale at α = 0.82, and showed that the reliability of these measurements had satisfactory measurement accuracy [45]. A value higher than 0.70 for Cronbach’s alpha reliability coefficient was considered acceptable [44,45]. Kirppendorf’s alpha statistic was used to assess the accuracy of the measurement scales [46]. Very good compliance of the PF_DP scale was achieved with 14 test items, good—3 test items; and for the P_DP scale, very good—11 test items, good—6 test items. The concordance of test items is very good when the score of this statistic is >0.8, good for 0.60–0.80, satisfactory for 0.40–0.59, and insufficient for <0.40 [46]. A Kirppendorf alpha coefficient of at least 0.40 was considered acceptable.

In the third stage, the survey was conducted in September/October 2024. A total of 250 questionnaires were distributed during this stage of the study. The criteria for inclusion in the study were age 60 and over, residence in the Lower Silesian Province, a lack of any occupational activity (retired, pensioners or without the right to benefits), and due to the methodological criteria of the International Physical Activity Questionnaire (Polish version, IPAQ, was used in the study), no illness, hospital stay, stay in rehabilitation, convalescence after illness and vacation/planned rest within 7 days before the survey [47]. Other criteria for exclusion from the study were missing questionnaires (16 cases) and respondents declaring that they did not consume any dairy products for medical or dietary reasons or of their own volition (4 cases). Ultimately, 230 questionnaires were included in the analyses. With the recruitment support of community organizations, the study was conducted in Wroclaw (32.6%, n = 75), Siechnice (23.5%, n = 54), Święta Katarzyna (20.1%, n = 46), Radwanice (7.8%, n = 18), Bogusławice (7.8%, n = 18), Żerniki Wrocławskie (4.3%, n = 10) and Duszniki Zdrój (3.9%, n = 9).

The study was conducted following the Declaration of Helsinki [48]. Participation in the survey was voluntary. Informed consent was obtained from all participants to participate in the study. The study was approved by the Rector’s Committee for Research Ethics of the University of Environmental and Life Sciences in Wroclaw on 12 February 2024, expressing a positive opinion on the compliance of the scientific research project with ethical principles (Resolution No. N0N00000.0011.1.2.3.2024).

### 2.2. Questionnaire

A proprietary questionnaire assessed taste preferences and the purchase frequency of selected food products. Eleven groups of typical dairy products were included, such as dairy fats (i.e., milk, including low-fat milk, condensed milk, powdered milk, dairy drinks, e.g., sweetened chocolate or fruit milk); fermented dairy drinks (e.g., yogurt, kefir, buttermilk, etc.); cottage cheese; grainy/rural cottage cheese; cottage cheese spreads for bread; yellow cheeses, including melted and molded cheeses (e.g., Gouda, Emmentaler, Podlaski, Camembert, Roquefort); butter (e.g., extra, cream); cream and sour cream; and 6 groups of functional dairy products, i.e., lactose-free (e.g., milk, cream cheese, etc.); cholesterol-lowering (e.g., “Benecol” yogurt); probiotic (e.g., “Activia” yogurt); enriched (e.g., yogurts with added calcium and/or vitamins); increased protein content (e.g., protein-rich milk drinks); and immune-boosting (e.g., “Actimel”). The frequency of consumption of these products was rated on a 6-point scale: 1—never; 2—once a month or less often; 3—several times a month; 4—once a week; 5—many times a week; and 6—daily. Concerning each food product, respondents expressed their attitude by marking their opinion on a 5-point scale, where 1—I like this food, it’s tasty; 2—I may or may not eat this food; 3—I don’t like this food, it’s unsavory; 4—I’ve never tried this food, but would try it if given the opportunity; and 5—I’ve never tried this food and don’t intend to try it. During the analysis, the opinion “I like this food; it is tasty” was coded as 1, while the other opinions were coded as 0. Respondents’ views on 11 groups of typical dairy products, including dairy fats, were then summed to create the variable “Taste preferences” (variable range 0–11).

To assess the level of physical activity, the full version of the International Physical Activity Questionnaire (Polish version) was used [47]. The full version of the IPAQ questionnaire consists of 5 independent parts. It includes detailed information on work-related physical activity, movement, household chores, recreation and sports, and time spent sitting or lying down [47,49,50]. General physical activity, including its movement components, housework, recreation, and sports, was assessed. Work-related activities were excluded. In addition, the activity of sitting or lying down was evaluated. Overall physical activity and its components were calculated using data on the number of days, duration in minutes per day, and MET values for different types of physical activity. The product and sum of these data allowed the assessment of physical activity in units of MET minutes/week, according to the methodological information of the IPAQ questionnaire [47]. Activity related to sitting or lying down was calculated in units of minutes/week based on the product of days and duration of activity related to sitting or lying down and summing their values [47].

Socio-demographic characteristics included gender, age, the place of residence, education, household composition, social activity, family relationships regarding economic factors, a subjective assessment of the household’s economic situation, and family and social financial support.

### 2.3. Statistical Analysis

Categorical variables are presented as a percentage of the sample (%), and continuous variables are presented as median and IQR. The Kolmogorov–Smirnov test was used to test the distribution of continuous variables (activity in MET-minutes/week, activity in minutes/week, and taste preferences). These variables did not have a normal distribution, so a non-parametric test (the Kruskal–Wallis test with Bonferroni correction) was used to compare mean ranks. For categorical variables, differences between groups were verified with the Chi-square test. A significance level of *p* < 0.05 was considered significant for both tests.

Based on the purchase frequency of 11 groups of typical dairy products, including dairy fats, factor analysis was conducted using principal component analysis (PCA) to identify patterns (factors) of buying behavior. Factor rotation was performed using an orthogonal (Varimax) transformation. The number of factors was determined using the following criteria: components with an eigenvalue ≥ 1, the scree plot test, and the interpretability of the factors. Variables (the frequency of purchase of particular food groups) were considered to load on the factor if the correlation coefficient took a value of at least 0.5. The selection of factors was confirmed through the Kaiser–Meyer–Olkin (KMO) measure of sampling adequacy and Bartlett’s sphericity test. Within each behavioral pattern (factor), 3 categories were created based on the tercile distribution of regression coefficients. The distinguished categories are 1st tercile (low pattern severity), 2nd tercile (moderate pattern severity), and 3rd tercile (high pattern severity). Statistical analysis was performed using IBM SPSS Statistics for Windows, version 29.0 (IBM Corp., Armonk, NY, USA).

## 3. Results

### 3.1. Characteristics of Study Sample

The characteristics of the study group, including socio-demographic characteristics and the variable describing financial situations, are presented in Table 1. The study group comprised 82.2% of women and 17.8% of men aged 60–91, with an average age of 70.6 years (SD ± 5.8). Most respondents had a high school education (53.0%), lived only with their partner (45.0%), and had a positively assessed financial situation (“We have enough money for basic needs, but we need to save for more serious purchases”—50.0%).

### 3.2. Buying Behavior Patterns

Based on the frequency of buying typical dairy products, including milk fats, three buying behavior patterns were distinguished using principal component analysis. The factor loading matrix for the identified buying behavior patterns (factors) is shown in Table 2. The KMO value was 0.794. Bartlett’s test showed significance at *p* < 0.001. The extracted buying behavior patterns explained 58.95% of the total variance (Table 2). The identified buying behavior patterns were named as follows: “conventional dairy products and fats” (factor 1), “powdered milk, condensed and unfermented dairy drinks” (factor 2), and “dairy fats” (factor 3).

The frequency of consumption of each group of dairy products is shown in Figure S1A–R in Supplementary Materials.

### 3.3. Buying Behavior Patterns and Their Relationship to Taste Preferences, Socioeconomic Characteristics, and Physical Activity

In the study group, taste preferences toward dairy products, which had a median of 7.0 (IQR = 2.0), differed only within the “conventional dairy products and fats” pattern. The higher the intensity of this pattern, the greater the taste preferences were recorded. In the other behavioral patterns, there were no differences in taste preferences after accounting for pattern severity (Table 3).

More people living with a family with and without a partner represented the second and third tercile of the “conventional dairy products and fats” pattern. In contrast, the most significant number of people living without a family or a partner (alone) represented the first tercile of this pattern (Table 4). In the case of the “dairy fats” pattern, the first tercile was represented by the most significant number of people who declared that they lived modestly or had a good or very good standard of living well. In contrast, the third tercile was represented by the most significant number of people declaring that they lived moderately (Table 4). Other socio-demographic and economic characteristics did not differentiate the adherence to the distinguished behavioral patterns concerning their tercile structure.

Respondents’ general physical activity varied only after accounting for the severity of the “conventional dairy products and fats” pattern, with significant differences observed between the first and third tercile of this pattern. Individuals representing the third tercile were characterized by higher physical activity than the first tercile of this pattern. No variation was shown in the other buying behavior patterns after accounting for general physical activity. The movement activity was higher in the third tercile of the “conventional dairy products and fats” pattern than in the second tercile. At the same time, an inverse relationship was observed in the “dairy fats” pattern. Activity related to housework was highest in the second tercile of the “powdered milk, condensed milk, and dairy drinks” pattern and, at the same time, significantly higher than in the first tercile. In contrast, activity related to sports and recreation was highest in the third tercile of the “conventional dairy products and fats” pattern while being only significantly higher than those representing the second tercile. Respondents’ lying or sitting activities showed no variation after considering the respondents’ affiliation with particular patterns of buying typical dairy products and fats (Table 5).

### 3.4. Buying Behavior Patterns and Their Relationship to Buying Patterns Regarding Functional Dairy Products

The frequency of buying functional dairy products and its relationship with adherence to identified buying behavior patterns is shown in Table 6. More than half of the respondents have never purchased functional dairy products. Most of the participants of the survey have never bought cholesterol-lowering dairy products (69.1%) and lactose-free products (66.1%).

Among those with a high intensity of the “conventional dairy products and fats” pattern (the third tercile), there were more people purchasing cholesterol-lowering, probiotic, mineral- and vitamin-enriched, protein-enhanced and immune-boosting dairy products at least once a week than in the first tercile of this pattern. In contrast, the first tercile had the highest number of never-buyers of probiotic dairy products, products enriched in minerals and vitamins, products with increased protein content, and immune-boosting products. The proportion of people who never bought cholesterol-lowering dairy products was similar in the first and third tercile (72.7 and 75.0, respectively). The frequency of purchasing lactose-free dairy products did not vary among the study group after considering the intensity of the “conventional dairy products and fats” and “powdered milk, condensed and unfermented dairy drinks” patterns. In the latter pattern, the purchase of probiotic dairy products was favored by higher intensity, with 11.8% in the first tercile and 26.0% in the third tercile. In contrast, the first tercile of this pattern had the highest number of people who had never purchased protein-enhanced dairy products (71.1%). In comparison, the third tercile had the lowest number of such people (48.1%) (Table 6).

Among those with a high intensity of the “dairy fats” pattern (the third tercile), there was the smallest number of people buying immune-boosting dairy products at least once a week (11.7%) compared to those in the first tercile (28.9%) and also the highest number of people who never bought such products (66.2%). In addition, the majority of people with a high intensity of this pattern declared that they did not buy lactose-free dairy products (76.6%). In comparison, the most significant number of people in the first tercile of this pattern consumed these products at least once a week (28.9%) (Table 6).

The frequency of buying functional dairy products did not vary for various socio-demographic and economic characteristics. However, the frequency of purchasing some products varied when physical activity was considered (Table 7). People buying lactose-free dairy products at least once a week were characterized by significantly higher physical activity than those buying these products less than once a week. Those who never bought immune-boosting dairy products were substantially less active in terms of moving around and performing chores around the house than those who bought such products at least once a week.

## 4. Discussion

This study evaluated the differentiation of the identified buying patterns in terms of taste preferences, selected socioeconomic characteristics, and level of physical activity. In addition, the frequency of purchasing buying functional products was compared, taking into consideration adherence to distinguished behavioral patterns and their selected determinants.

Three buying behavior patterns were identified in the study. The “typical” dairy product pattern was described as “conventional products and dairy fats”. This pattern included milk, fermented milk beverages, cottage cheese and curds, yellow cheese, butter, cream, and sour cream. Previous studies have reported that these are products frequently purchased and consumed by older people in Poland [35]. In general, in recent years, the consumption of dairy products in the Polish population has increased. For example, milk consumption, including milk intended for producing dairy products, excluding butter, increased from 189 L per person in 2010 to 276 L per person in 2023. In the case of butter, the increase was from 4.3 kg to 5.9 kg, respectively [50,51,52]. Only milk and sour cream consumption has decreased over the last 10 years [25]. The report entitled “The Milk Market-Status and Prospects” showed that pensioner households in Poland, compared to other households, consumed more milk, cheese, cottage cheese, yogurt, and cream in 2023 [51]. Butter, cream, and sour cream, which are included in the “dairy fats” pattern, are also products commonly purchased and consumed by this age group [35], and the highest consumption was recorded for sour cream [32]. The high interest in purchasing typical dairy products such as milk, butter, and sour cream among older people may result from their attachment to traditional Polish cuisine based on those products. Previous Polish studies reported that the “traditional Polish” dietary pattern is characteristic for older adults. However, this pattern was more common among older adults with a lower socioeconomic status, living with their family and of better health [53,54,55]. Sensory preferences, formed based on presence of such a pattern in childhood, were also crucial [56]. Sensory preferences and living with family influence older people’s purchasing behavior toward typical dairy products, which was confirmed in this study. The identification of the “milk powder, condensed and unfermented milk drinks” pattern was not expected prior to conducting the survey. The literature says little about older adults purchasing and consuming such dairy products. Also, reports based on data from the Central Statistical Office in Poland on the milk market do not indicate a significant amount of purchases and the consumption of such dairy products among older people [25,32,51,52,53]. Little is also known about the factors that differentiate their consumption or purchase. Our study did not identify any socioeconomic factor differentiating purchasing behavior within this pattern, but it showed that housework-related activity was greater at its moderate intensity. This may be because dairy products characteristic for this pattern are commonly used to prepare dishes and desserts that require effort at home.

The findings of this study showed that more than half of the respondents had never purchased functional dairy products, with the highest number of people having never purchased cholesterol-lowering dairy products (69.1%) and lactose-free products (66.1%). Even though dairy products are the dominant group of functional products in the food market [57] and they are most commonly associated with this food group [58], the level of acceptance of these products is still low [59]. Older consumers approach them with great skepticism [58]. The low level of acceptance of lactose-free dairy products has been shown among the elderly [60], which may limit the likelihood of purchasing this food group, even though it is mainly dedicated to the elderly, due to frequent lactose intolerance. An earlier study in a group of German consumers reported that margarine or fat blends and dairy products that lower blood cholesterol were preferred among older adults [61], but this was not confirmed in our study. In contrast, a high preference was shown for functional calcium-enriched dairy products [62]. This may be due to the high level of knowledge regarding the importance of calcium-rich dairy products in preventing osteoporosis, especially among older women [59]. The high intensity of the “conventional dairy products and fats” pattern was associated with more frequent purchases (at least once a week) of many functional dairy products. This result may suggest that the pro- or anti-purchase behavior of older people in the dairy market applies to most products uniformly and not only to selected groups.

The purchase of most dairy products is routine due to the high frequency of consumption and the constant nature of the needs they satisfy [38]. Among the top factors considered when purchasing dairy products in Poland are shelf life [34,37,38], price, and brand [34], but also sensory impressions, especially taste and smell [36,38]. The present study also reported the association between taste preferences (“I like this food, it’s tasty”) and high intensity (the third tercile) of the “conventional dairy products and fats” pattern. Previous studies have reported that the elderly’s response, “I like this food a lot; it’s tasty”, referred mostly to natural yogurt, buttermilk, and butter [60], which may confirm the association of taste preferences with this buying behavior pattern. No relationship was shown between taste preferences and the intensity of other patterns, even though, in another study, butter had a high preference level among older people [60]. This may explain why butter and cream were often bought [32,35].

Financial situation differentiates the consumption of dairy products in Polish households, with the highest consumption of these products in households with the highest income [35]. However, the study’s results did not confirm the differences in the purchase of “conventional products and dairy fats” in view of the self-assessment of the financial situation. At the same time, there were differences in the “dairy fats” pattern. Most respondents in the third tercile of this pattern declared that they “have an average standard of living”. Murawska’s research [35] showed that the highest consumption of butter and cream was observed in the households of pensioners but did not show any differentiation of purchasing behaviors related to this group of products in terms of the income level. However, it was noted that the financial resources or income of the elderly households were the strongest determinants differentiating the level of milk consumption. On the other hand, the level of yogurt and cheese consumption was most strongly differentiated by the income and composition of the elderly’s households [35]. In European countries, income was found to be less important in differentiating the consumption behavior of dairy products [63]. The most significant differences in dairy consumption by income were observed in Middle Eastern, Asian, African, and South American countries [64].

Older respondents living with their family, both with and without a partner, represented the second and third tercile of the “conventional dairy products and fats” pattern. In contrast, living alone (without family or partner) was associated with this pattern’s low intensity (the first tercile). Many studies suggest that family size affects the increase in dairy consumption [64,65]. This is due to the perception of dairy products as essential components of the diet and the presence of children [66]. While the overall consumption of milk and milk products in large families is increasing per family member, this is no longer so evident [64,67,68]. As family size increases, the consumption of dairy products per family member may decrease. The problem arises mainly in families with the lowest incomes, where there is a decline in the consumption of dairy products per family member and their overall consumption [68,69]. This may suggest that the higher intensity of the “conventional dairy products and fats” pattern among older adults living with their families is not necessarily associated with higher consumption. In addition, a study by Roustaee et al. [64] found that the presence of older family members (60+) in the household was associated with lower yogurt and cheese consumption.

A Polish study showed that older people tend to be in the upper tercile of the “healthy eating and high physical activity” pattern, which is conducive to their better health [40]. At the same time, other studies showed that low physical activity was associated with a low intake of milk and dairy products and vice versa, although these studies usually involved children [40,42,70]. However, higher physical activity among older adults was also associated with a dietary pattern based on dairy products [71,72]. In addition to the inadequate consumption of milk and dairy products, low physical activity is also the reason for the lower consumption of legumes, fruits, meat, vegetables, and cereals [40]. While studies indicate an association of dairy product consumption with physical fitness in older people [16], no studies indicate an association between the purchase frequency of these products and physical activity. Our study showed that lower overall physical activity and activity related to movement, sports, and recreation were accompanied by a lower intensity of the “conventional dairy products and fats” pattern. This relationship may result from the poorer physical condition of people who consume small amounts of dairy products. For example, from a prospective study in the Spanish Senior-ENRICA cohort, it is known that the consumption of seven or more servings of low-fat dairy products, particularly low-fat milk per week versus less than one serving, was associated with a lower incidence of frailty syndrome, including loss of physical function [15,21,73]. Moreover, it was observed that older people in the highest tercile, compared to those in the lowest tercile of dairy consumption, had significantly higher grip strength and a lower probability of poor performance in the standing up and walking time trials, while no differences were observed in the incidence of falls [18]. In further studies, it is necessary to jointly examine the relationship between physical activity and milk and dairy consumption and between physical activity and the purchase of these products. This is particularly important in the group of older people, whose milk and dairy consumption may be lower due to difficulties in making food purchases.

In Polish studies, the region (voivodeship) and place of residence (city or village) were shown to have little significance in differentiating the consumption of dairy products among older people [35]. This study also did not show such differentiation. To our knowledge, the relationships between purchasing behavior related to milk and dairy products, physical activity, taste preferences and socioeconomic characteristics of older Poles have not been comprehensively studied. Therefore, these results complement the knowledge in this field and can be used in various activities aimed at older people. The obtained results suggest, however, that further research should continue to pay attention to preferences, economic situation, family structure, and physical activity as key factors in determining the purchase of typical dairy products. It is also critical to pursue in-depth assessments of why older people are reluctant to purchase functional dairy products, the properties of which could positively influence evolutionary changes and the health of older people.

### Strengths and Limitations

The strength of this study is its comprehensive approach to the assessment of older consumers’ purchasing behavior in the form of distinguishing behavioral patterns. This allows for a more comprehensive outlook on the behaviors related to purchasing dairy products. Therefore, the characteristics of older people who purchase dairy products with different intensity, additionally supplemented with the frequency of purchase of functional dairy products, may find practical use in the dairy industry, both in developing marketing strategies and developing new products addressed to older people. This study investigated the frequency of buying conventional dairy products, which may express the habitual behavior of older people, referring to the actions generated by the habitual process [74]. According to the Habit Theory [75], habits are acquired slowly through the repetition of an action in stable circumstances and may hinder the emergence of new behaviors, such as buying functional foods. This is because a habit is associated with a permanent cognitive orientation, which is called “habitual mindset”, which makes the individual less attentive to new information and directions of action, and thus contributes to the maintenance of habitual behavior [75,76], as is the case of dairy products regarding the lower inclination to purchase functional foods.

This study has some limitations that should be mentioned. Firstly, the study is a cross-sectional one, so assessing the direction of influence and causal relationship between variables is impossible. In this situation, it is difficult to determine whether, for example, the level of physical activity influences consumer behavior according to a specific pattern or whether a specific pattern of consumer behavior influences the level of physical activity. Objectively, the direction of influence can be inferred based on longitudinal studies, e.g., prospective cohort studies or studies based on analysis using structural equation modeling. In addition, the non-probabilistic sampling method did not allow a representative sample to emerge. Moreover, the overrepresentation of female respondents may have unexpectedly affected the relationship between these determinants and purchasing behavior. The lack of a representative survey sample and the technical support of professional opinion polling companies limited the ability to reach elderly people of low socioeconomic status and the socially excluded, which may have been the reason for the lack of the identification of statistically significant differences affecting the model of consumer behavior and its determinants.

## 5. Conclusions

The buying behaviors of older people in the dairy market appears to be related to taste preferences, household structure, the self-assessment of the household’s financial situation, and the level of physical activity. These relationships do not apply to all dairy product groups in the same way. The high intensity of the “conventional dairy and fat” pattern was associated with higher taste preferences for dairy products, living with a family (with or without a partner), and higher physical activity, including mobility-related activities, as well as sports and recreation. In addition, the high intensity of the “milk fat” pattern was associated with moderate movement activity and with a financial situation described as “we have an average standard of living”. The moderate intensity of the “milk powder, condensed and unfermented milk drinks” pattern was accompanied by a higher amount of housework-related activity. The respondents were characterized by low interest in purchasing functional dairy products. The high intensity of the “conventional dairy products and fats” pattern was accompanied by more frequent purchases (at least once a week) of many products from the functional dairy product group. In addition, higher physical activity correlated with a higher frequency of purchasing lactose-free and immune-boosting dairy products. Considering the limitations of the study, further research is needed to assess the association between socio-demographic and economic characteristics, taste preferences, physical activity and the behavior of older people in the dairy market. To determine the causal relationship between buying behaviors and its determinants, it is vital to conduct prospective cohort studies in a representative group of older people. This will help determine the dairy industry’s marketing strategies and forms of effective education for older consumers.

## Figures and Tables

**Table 1:** Characteristics of the study group.

Variables	Categories of Variables	% (N)
Gender	women	82.2 (89)
	men	17.8 (41)
Age	60–70 years	51.7 (119)
	71–80 years	44.3 (102)
	over 80 years	4.0 (9)
Place of residence	village	37.0 (85)
	city with <100,000 inhabitants	27.8 (64)
	city with >100,000 inhabitants	35.2 (81)
Education	elementary	2.6 (6)
	basic vocational	15.7 (36)
	secondary	53.0 (122)
	higher	28.7 (66)
Household composition	I live alone	33.5 (77)
	I live with my partner	45.2 (104)
	I live with my family and partner	10.9 (25)
	I live with my family without a partner	10.4 (24)
Self-assessment of financial situation	We live modestly—we have to be very frugal daily	11.7 (27)
	We have an average standard of living—we have enough money for basic needs, but we need to save for more serious purchases	50.0 (115)
	We have a good standard of living—we can afford a lot without special saving	30.9 (71)
	We live a very good standard of living—we can afford certain luxuries	7.4 (17)

**Table 2:** Factor loadings matrix for buying behavior patterns (factors) identified based on principal component analysis.

Dairy Products	Buying Behavior Patterns (Factors)		
	1 *	2	3
Milk, including low-fat milk	0.566	0.127	0.113
Fermented dairy drinks, such as yogurt, kefir, buttermilk, etc.	0.697	−0.106	0.043
Cottage cheese	0.721	−0.071	0.201
Grainy/rural cottage cheese	0.720	0.060	−0.072
Cottage cheese spread on bread	0.722	0.062	−0.113
Yellow cheeses, including processed and moldy cheese (e.g., Gouda, Emmentaler, Podlaski, Camembert, Roquefort, etc.)	0.649	0.135	0.041
Butter (e.g., extra or cream butter)	0.543	0.121	0.562
Cream and sour cream	0.551	0.135	0.625
Condensed milk	0.182	0.788	0.011
Milk powder	−0.071	0.881	−0.016
Dairy drinks such as sweetened chocolate or fruit milk	0.023	0.858	−0.048
% of variance explained	31.54	18.25	9.15
Total explained variance (%)	58.95		

* 1—“conventional dairy products and fats” pattern, 2—“powdered milk pattern, condensed and unfermented dairy drinks”, 3—“dairy fats” pattern.

**Table 3:** Characteristics of buying behavior patterns in terms of taste preferences (median; IQR).

Buying Behavior Patterns (Factors)	Taste Preferences		
	1st Tercile	2nd Tercile	3rd Tercile
“Conventional dairy products and fats” patterns —Factor 1 (*p* < 0.001)	6.0 a *; 3.5	7.0 a; 2.0	8.0 a; 2.0
“Powdered milk, condenses and unfermented dairy drinks” patterns—Factor 2 (*p* = 0.238)	7.0; 2.0	7.0; 3.0	7.0; 3.0
“Dairy fats” patterns—Factor 3 (*p* = 0.062)	7.5; 2.0	8.0; 3.0	7.0; 3.0

* Values marked with the same letters significantly differ (*p* < 0.05), Kruskal–Wallis test with Bonferroni correction.

**Table 4:** Characteristics of buying behavior patterns, taking into account selected socioeconomic characteristics.

Variables	Categories of Variables	Total % (N)	Buying Behavior Patterns (Factors) % (N)		
			1st Tercile	2nd Tercile	3rd Tercile
“Conventional dairy products and fats” pattern—Factor 1 (*p* = 0.002 *)					
Household composition	I live alone	33.5 (77)	51.9 (40)	24.7 (19)	23.7 (18)
	I live with my partner	45.2 (104)	36.4 (28)	50.6 (39)	48.7 (37)
	I live with my family and partner	10.9 (25)	9.1 (7)	11.7 (9)	11.8 (9)
	I live with my family without a partner	10.4 (24)	2.6 (2)	13.0 (10)	15.8 (12)
“Dairy fats” pattern—Factor 3 (*p* = 0.018 *)					
Self-assessment of respondent’s financial situation	We live modestly—we have to be very frugal daily	11.7 (27)	14.5 (11)	9.1 (7)	11.7 (9)
	We have an average standard of living—we have enough money for our daily needs, but we need to save for more serious purchases	50.0 (115)	32.9 (25)	54.5 (42)	62.3 (48)
	We have a good standard of living—we have enough money for a lot without special saving	30.9 (71)	42.1 (32)	29.9 (23)	20.8 (16)
	We have a very good standard of living—we can afford certain luxuries	7.4 (17)	10.5 (8)	6.5 (5)	5.2 (4)

N—number of people; * significance level, Chi^2^ test.

**Table 5:** Characteristics of buying behavior patterns, including physical activity (median; IQR).

Buying Behavior Patterns (Factors)	Buying Behavior Patterns (Factors)			Total
	1st Tercile	2nd Tercile	3rd Tercile	
General physical activity (MET minutes/week)				
“Conventional dairy products and fats” patterns—Factor 1 (*p* = 0.004)	4341.0 a *; 3118.5	4938.0; 4265.5	5979.5 a; 5868.0	4931.3; 4413.4
“Powdered milk, condensed and unfermented dairy drinks”—Factor 2 (*p* = 0.061)	4563.0; 4065.0	5862.0; 5337.0	4620.0; 3546.5	
“Dairy fats”—Factor 3 (*p* = 0.183)	4984.5; 4124.3	5265.0; 4566.0	4530.0; 4519.0	
Movement activity (MET minutes/week)				
“Conventional dairy products and fats” patterns—Factor 1 (*p* = 0.028)	1386.0; 1565.5	1582.5 a; 1633.5	1803.0 a; 4015.1	1552.0; 1650.5
“Powdered milk, condensed and unfermented dairy drinks”—Factor 2 (*p* = 0.523)	1543.5; 1619.0	1550.0; 1968.0	1653.0; 1709.5	
“Dairy fats”—Factor 3 (*p* = 0.004)	1833.0; 1672.1	1788.0 a; 2594.0	1377.0 a; 1119.0	
Activity related to work at home (MET minutes/week)				
“Conventional dairy products and fats” patterns—Factor 1 (*p* = 0.209)	1575.0; 2070.0	1640.0; 2050.0	1965.0; 2662.5	1680.0; 2115.0
“Powdered milk, condensed and unfermented dairy drinks”—Factor 2 (*p* = 0.001)	1440.0 a; 1620.0	1895.0 a; 3330.0	1770.0; 1935.0	
“Dairy fats”—Factor 3 (*p* = 0.330)	1770.0; 2152.5	1980.0; 2162.5	1470.0; 2162.5	
Activity related to sports and recreation (MET minutes/week)				
“Conventional dairy products and fats” patterns—Factor 1 (*p* = 0.017)	1320.0; 2249.3	1314.0 a; 1699.5	1908.0 a; 2193.3	1590.0; 1941.0
“Powdered milk, condensed and unfermented dairy drinks”—Factor 2 (*p* = 0.406)	1662.0; 2232.0	1785.0; 1715.0	1386.0; 2018.3	
“Dairy fats”—Factor 3 (*p* = 0.435)	1611.0; 1759.5	1737.0; 2272.5	1386.0; 1771.0	
Activity associated with sitting or lying down (minutes/week)				
“Conventional dairy products and fats” patterns—Factor 1 (*p* = 0.515)	630.0; 540.0	690.0; 540.0	625.0; 660.0	660.0; 615.0
“Powdered milk, condensed and unfermented dairy drinks”—Factor 2 (*p* = 0.744)	675.0; 630.0	680.0; 700.0	660.0; 540.0	
“Dairy fats”—Factor 3 (*p* = 0.417)	540.0; 780.0	660.0; 540.0	690.0; 495.0	

* Values marked with the same letters significantly differ (*p* < 0.05), Kruskal–Wallis test with Bonferroni correction.

**Table 6:** Frequency of purchases of functional dairy products in view of adherence to identified buying behavior patterns.

Functional Dairy Products	Buying Frequency	Total	“Conventional Dairy Products and Fats” Pattern			“Powdered Milk, Condensed and Unfermented Dairy Drinks” Pattern			“Dairy Fats” Pattern		
			1st Tercile	2nd Tercile	3rd Tercile	1st Tercile	2nd Tercile	3rd Tercile	1st Tercile	2nd Tercile	3rd Tercile
Lactose-free	never	66.1 (152)	66.2 (51)	64.9 (50)	67.1 (51)	64.5 (49)	70.1 (54)	63.6 (49)	53.9 (41)	67.5 (52)	76.6 (59)
	less than once a week	18.7 (43)	23.4 (18)	20.8 (16)	11.8 (9)	19.7 (15)	18.2 (14)	18.2 (14)	22.4 (17)	23.4 (18)	10.4 (8)
	at least once a week	15.2 (35)	10.4 (8)	14.3 (11)	21.1 (16)	15.8 (12)	11.7 (9)	18.2 (14)	23.7 (18)	9.1 (7)	13.0 (10)
	*p*-value (Chi^2^ test)		*p* = 0.204			*p* = 0.836			*p* = 0.011		
Lowering cholesterol levels	never	69.1 (159)	72.7 (56)	59.7 (46)	75.0 (57)	71.1 (54)	77.9 (60)	58.4 (45)	65.8 (50)	67.5 (52)	74.0 (57)
	less than once a week	16.5 (38)	16.9 (13)	26.0 (20)	6.6 (5)	13.2 (10)	13.0 (10)	23.4 (18)	15.8 (12)	20.8 (16)	13.0 (10)
	at least once a week	14.3 (33)	10.4 (8)	14.3 (11)	18.4 (14)	15.8 (12)	9.1 (7)	18.2 (14)	18.4 (14)	11.7 (9)	13.0 (10)
	*p*-value (Chi^2^ test)		*p* = 0.018			*p* = 0.097			*p* = 0.520		
Probiotic	never	51.3 (118)	66.2 (51)	40.3 (31)	47.4 (36)	55.3 (42)	62.3 (48)	36.4 (28)	43.4 (33)	53.2 (41)	57.1 (44)
	less than once a week	30.4 (42)	26.0 (20)	40.3 (31)	25.0 (19)	32.9 (25)	20.8 (16)	37.7 (20)	32.9 (25)	32.5 (25)	26.0 (25)
	at least once a week	18.3 (42)	7.8 (6)	19.5 (15)	27.6 (21)	11.8 (9)	16.9 (13)	26.0 (20)	23.7 (18)	14.3 (11)	16.9 (13)
	*p*-value (Chi^2^ test)		*p* = 0.002			*p* = 0.009			*p* = 0.377		
Enriched with vitamins and minerals	never	50.0 (115)	58.4 (45)	41.6 (32)	50.0 (38)	59.2 (45)	51.9 (40)	39.0 (30)	48.7 (37)	45.5 (35)	55.8 (43)
	less than once a week	32.2 (74)	31.2 (24)	40.3 (31)	25.0 (19)	25.0 (19)	32.5 (25)	39.0 (30)	32.9 (25)	36.4 (28)	27.3 (21)
	at least once a week	17.8 (41)	10.4 (8)	18.2 (14)	25.0 (19)	15.8 (12)	15.6 (12)	22.1 (17)	18.4 (14)	18.2 (14)	16.9 (13)
	*p*-value (Chi^2^ test)		*p* = 0.048			*p* = 0.151			*p* = 0.747		
Protein-rich	never	60.9 (140)	70.1 (54)	51.9 (40)	60.5 (46)	71.1 (54)	63.6 (49)	48.1 (37)	59.2 (45)	59.7 (46)	63.6 (49)
	less than once a week	23.9 (55)	23.4 (18)	32.5 (25)	15.8 (12)	13.2(10)	23.4 (18)	35.1 (27)	26.3 (20)	27.3 (21)	18.2 (14)
	at least once a week	15.2 (35)	6.5 (5)	15.6 (12)	23.7 (18)	15.8 (12)	13.0 (10)	16.9 (13)	14.5 (11)	13.0 (10)	18.2 (14)
	*p*-value (Chi^2^ test)		*p* = 0.007			*p* = 0.021			*p* = 0.649		
Immune-enhancing	never	52.2 (120)	64.9 (50)	39.0 (30)	52.6 (40)	57.9 (44)	57.1 (44)	41.6 (32)	39.5 (30)	50.6 (39)	66.2 (51)
	less than once a week	28.3 (65)	23.4 (18)	42.9 (33)	18.4 (14)	26.3 (20)	24.7 (19)	33.8 (26)	31.6 (24)	31.2 (24)	22.1 (17)
	at least once a week	19.6 (45)	11.7 (9)	18.2 (14)	28.9 (22)	15.8 (12)	18.2 (14)	24.7 (19)	28.9 (22)	18.2 (14)	11.7 (9)
	*p*-value (Chi^2^ test)		*p* < 0.001			*p* = 0.245			*p* = 0.012		

**Table 7:** Frequency of buying functional dairy products in view of physical activity (median; IQR).

Functional Dairy Products	Frequency of Buying			Total
	Never	Less than Once a Week	At Least Once a Week	
Lactose-free	General physical activity (MET-minutes/week) *p* = 0.018			
	4776.0; 3874.1	4608.0 a *; 4128.0	7840.0 a; 6192.0	4931.3; 4413.0
Immune-enhancing	Movement activity (MET-minutes/week) *p* = 0.020			
	1416.0 a; 1400.3	1614.0; 1617.0	2185.0 a; 2274.0	1552.0; 1655.5
Immune-enhancing	Activity related to work at home (MET-minutes/week) *p* = 0.050			
	1500.0 a; 2302.5	1640.0; 1747.5	2250.0 a; 2175.0	1680.0; 2115.0

* Values marked with the same letters significantly differ (*p* < 0.05), Kruskal–Wallis test with Bonferroni correction.

## Data Availability

Data presented in this study are available on request from the corresponding author. The data are not publicly available due to the ongoing study.

## Supplementary Materials

The following supporting information can be downloaded at: https://www.mdpi.com/article/10.3390/nu17071127/s1.
