# Supplementary material for: Physical Activity, Taste Preferences, Selected Socioeconomic Characteristics: Differentiators of Consumer Behavior Among Older Adults in the Dairy Market in Poland—A Pilot Study
Source: Nutrients. 2025 Mar 24;17(7):1127. doi: 10.3390/nu17071127 (PMC11990365; doi:10.3390/nu17071127)
Supplement: Supplementary file 1 [file nutrients-17-01127-s001.zip › nutrients-3528643-supplementary.pdf]

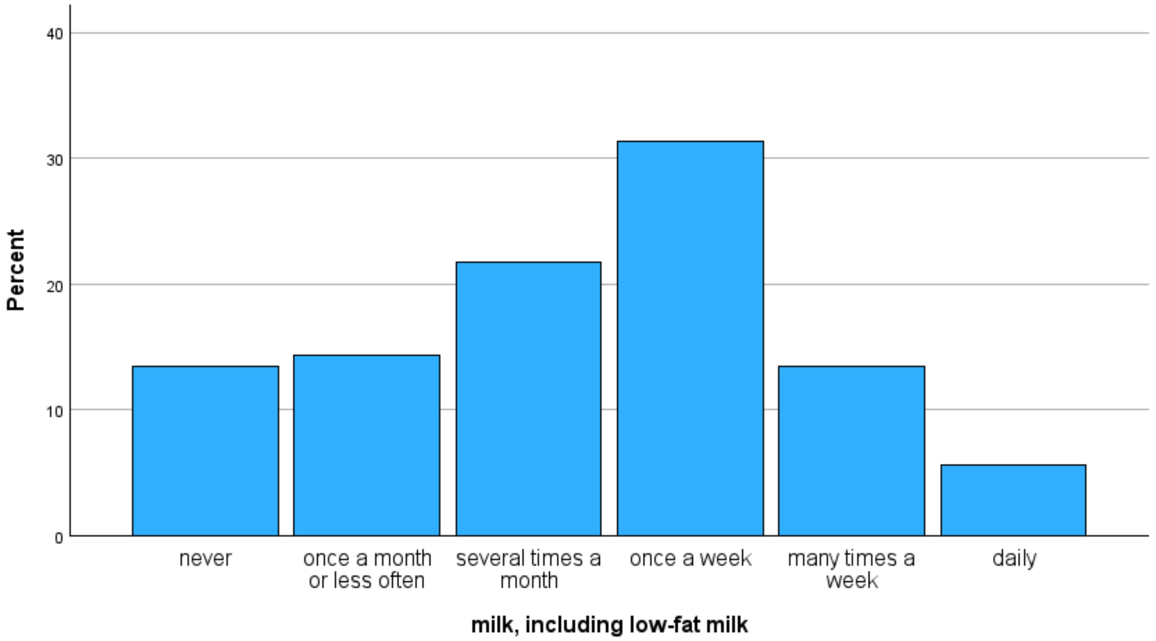

Figure S1A. Frequency of purchase of milk, including low-fat milk

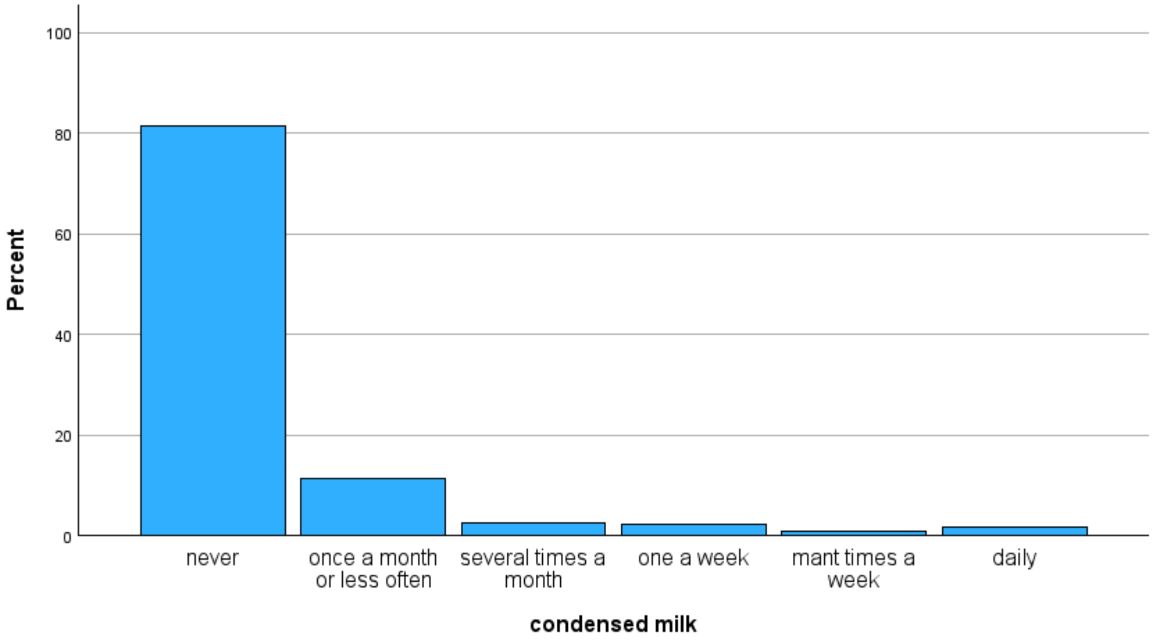

Figure S1B. Frequency of purchase of condensed milk

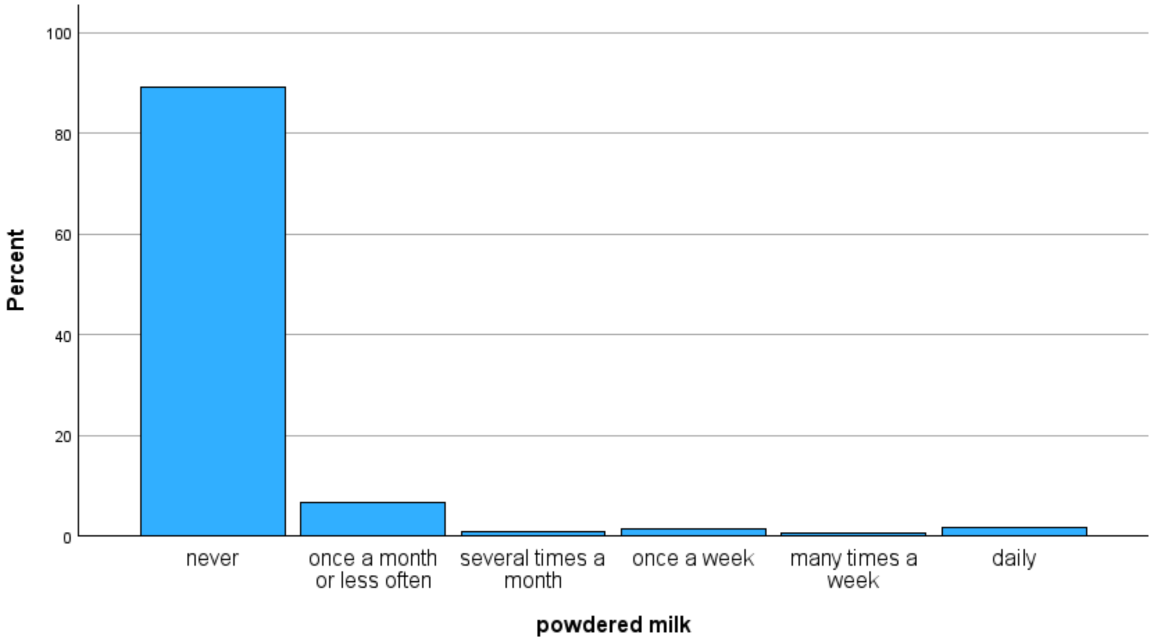

Figure S1C. Frequency of purchase of powdered milk

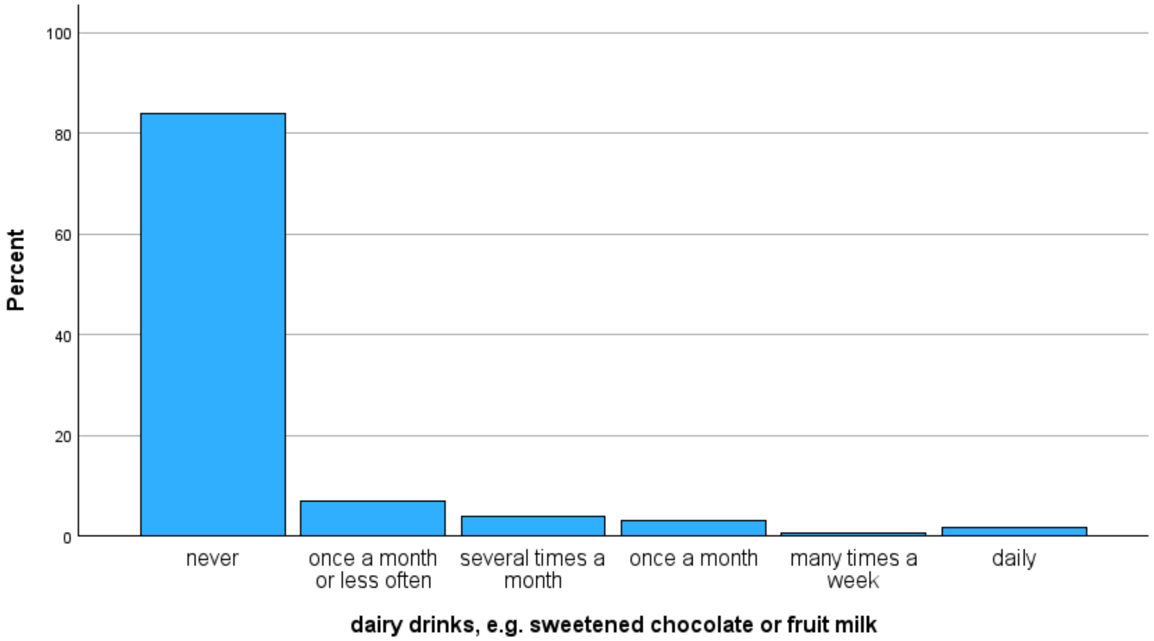

Figure S1D. Frequency of purchase of dairy drinks, e.g., sweetened chocolate or fruit milk

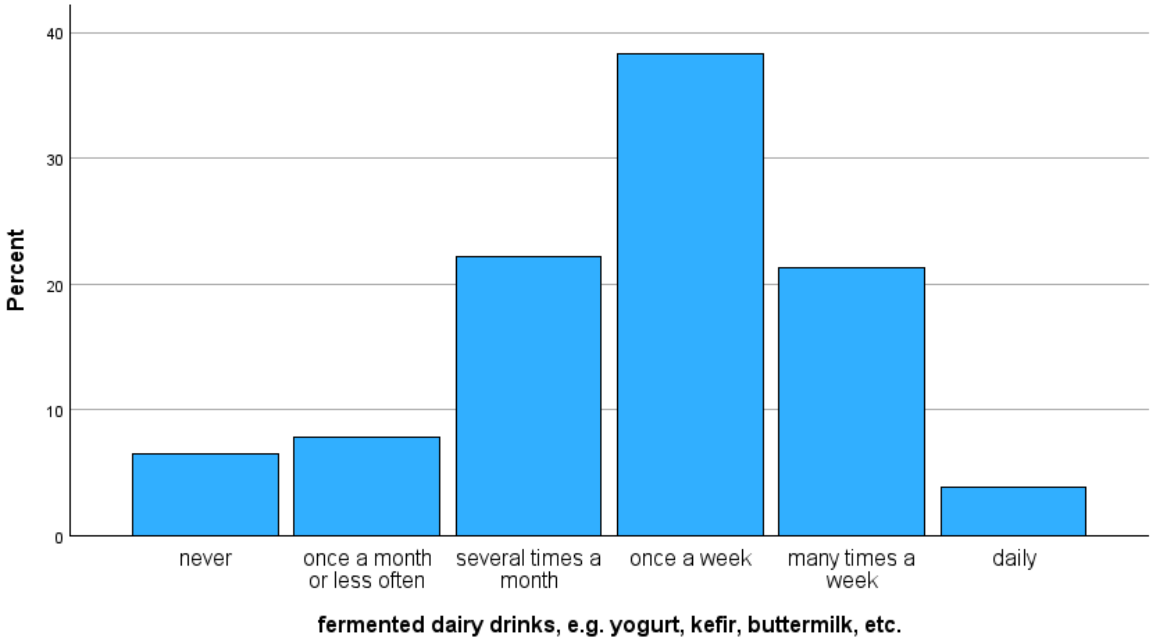

Figure S1E. Frequency of purchase of fermented dairy drinks, e.g., yogurt, kefir, buttermilk, etc.

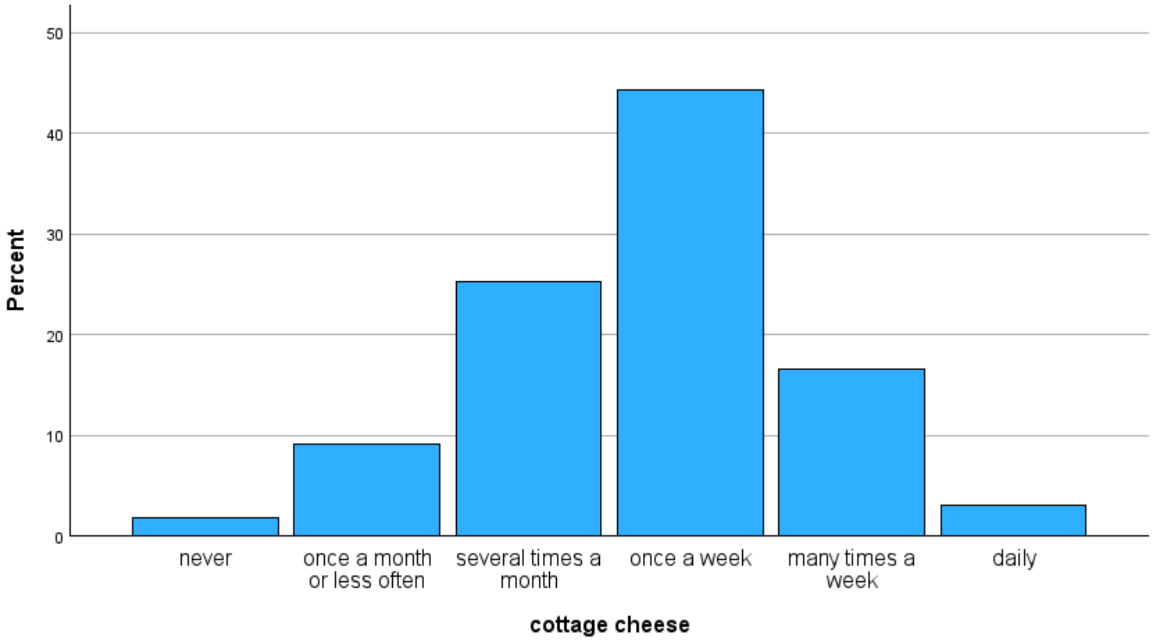

Figure S1F. Frequency of purchase of cottage cheese

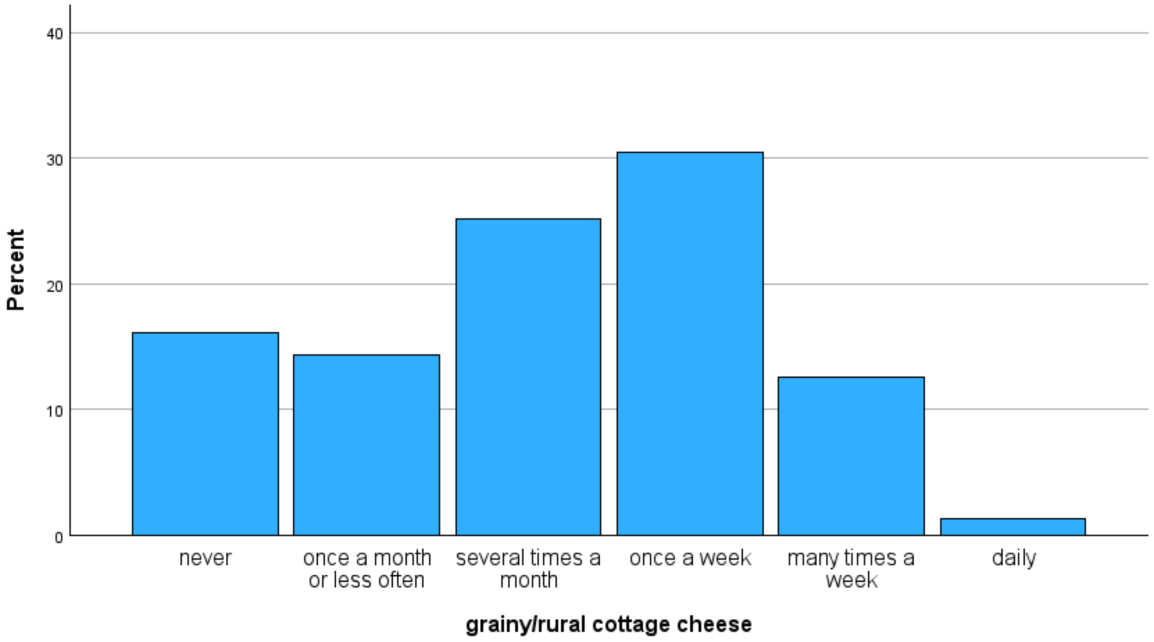

Figure S1G. Frequency of purchase of grainy/rural cottage cheese

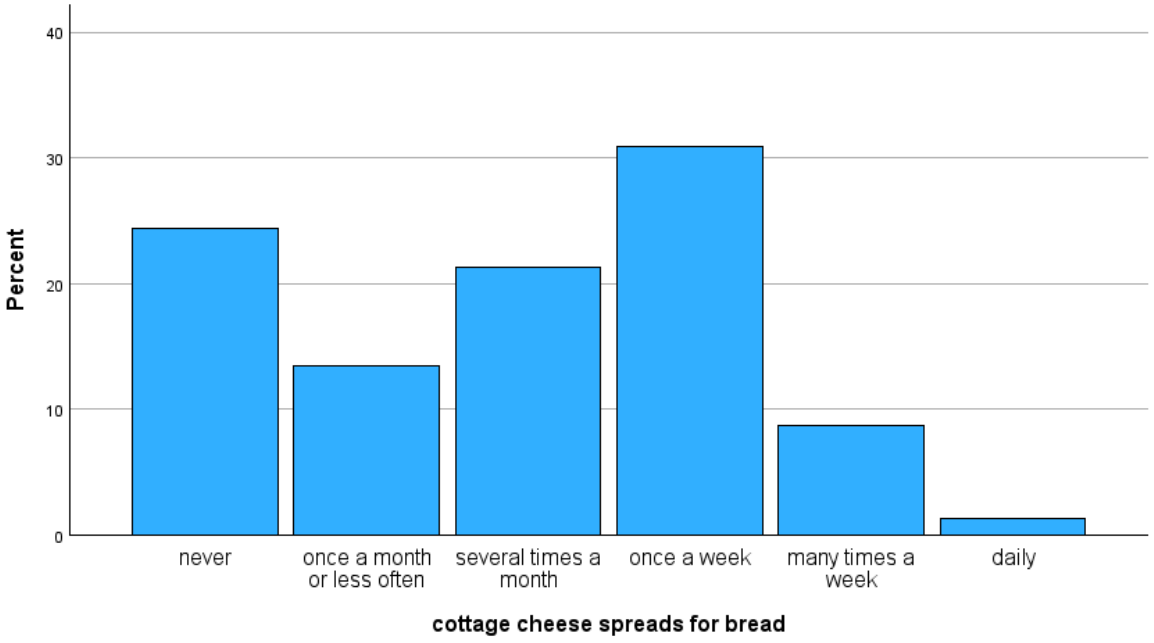

Figure S1H. Frequency of purchase of cottage cheese spreads for bread

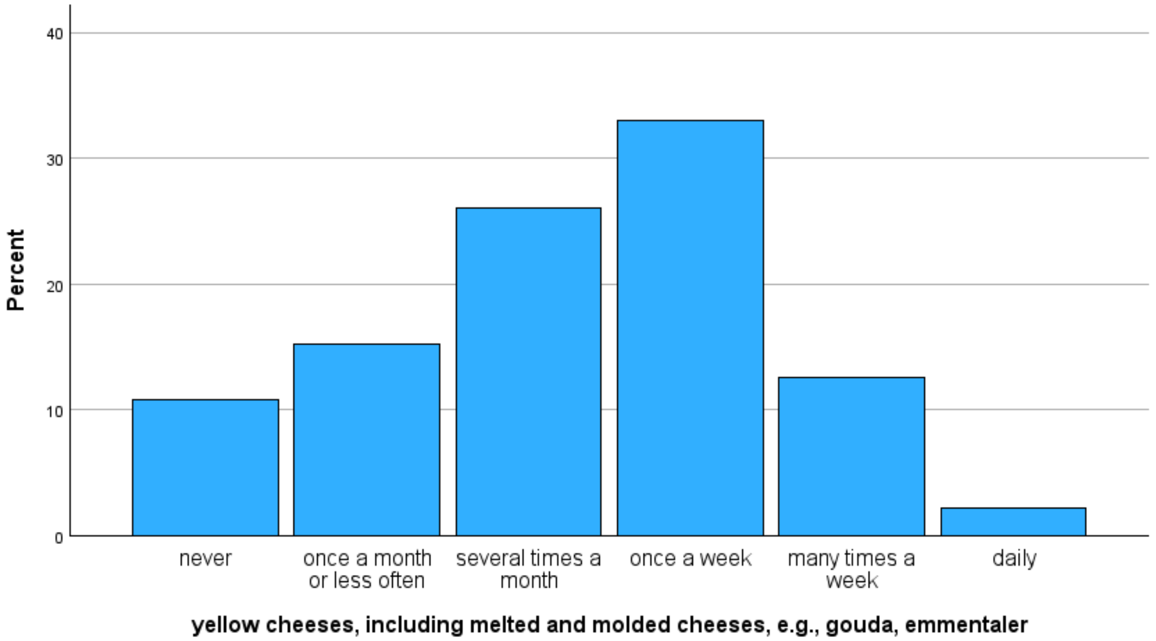

Figure S1I. Frequency of purchase of yellow cheeses, including melted and molded cheeses

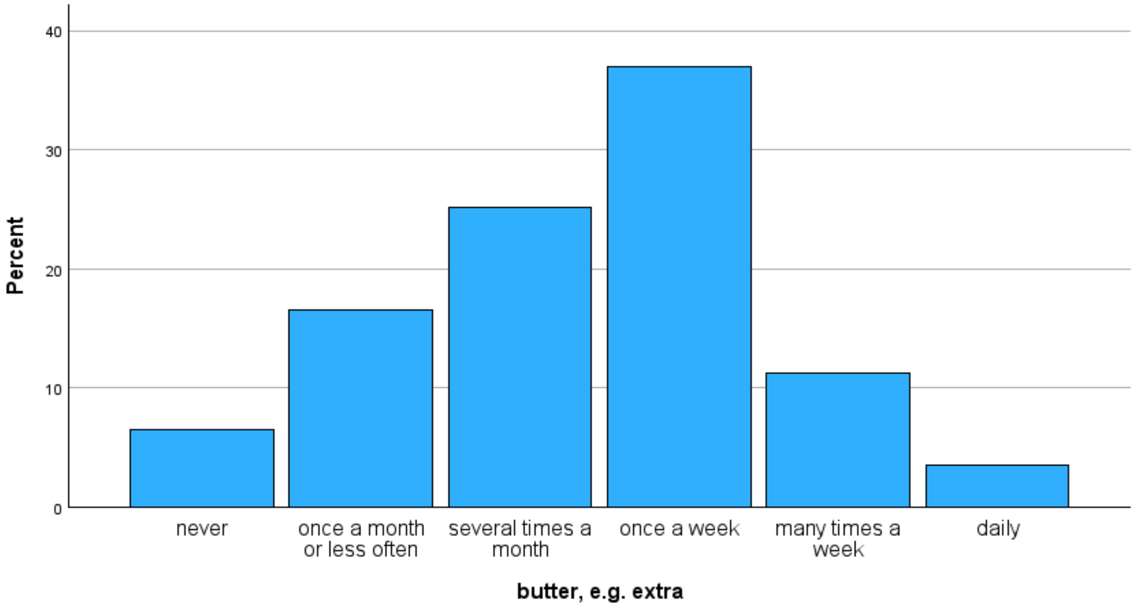

Figure S1J. Frequency of purchase of butter

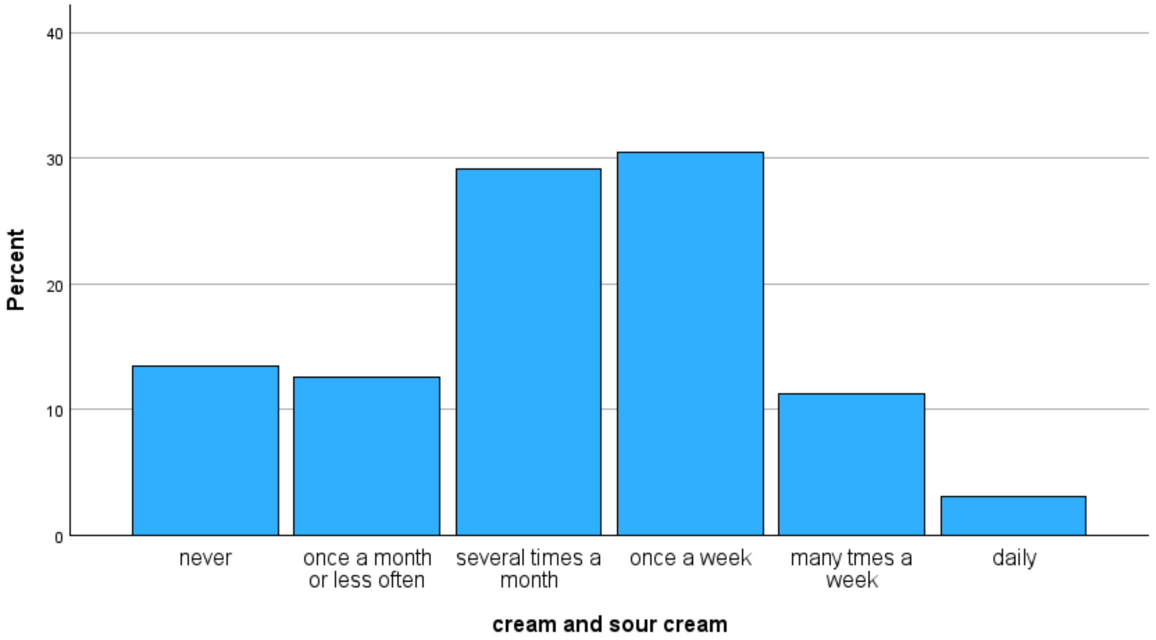

Figure S1K. Frequency of purchase of cream and sour cream

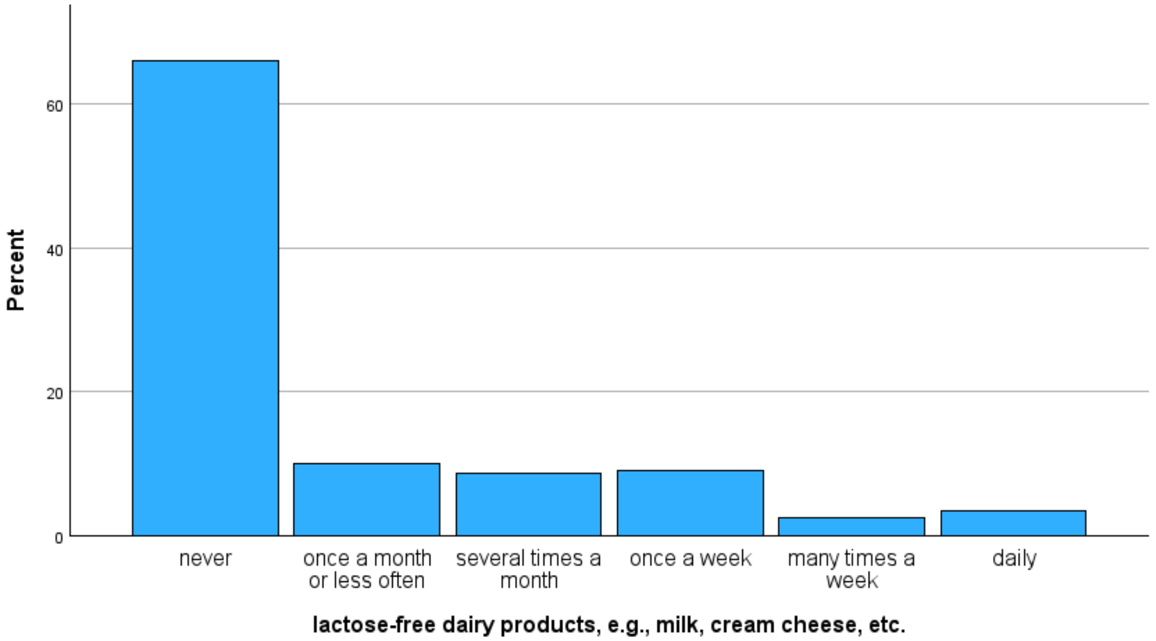

Figure S1L. Frequency of purchase of lactose-free dairy products

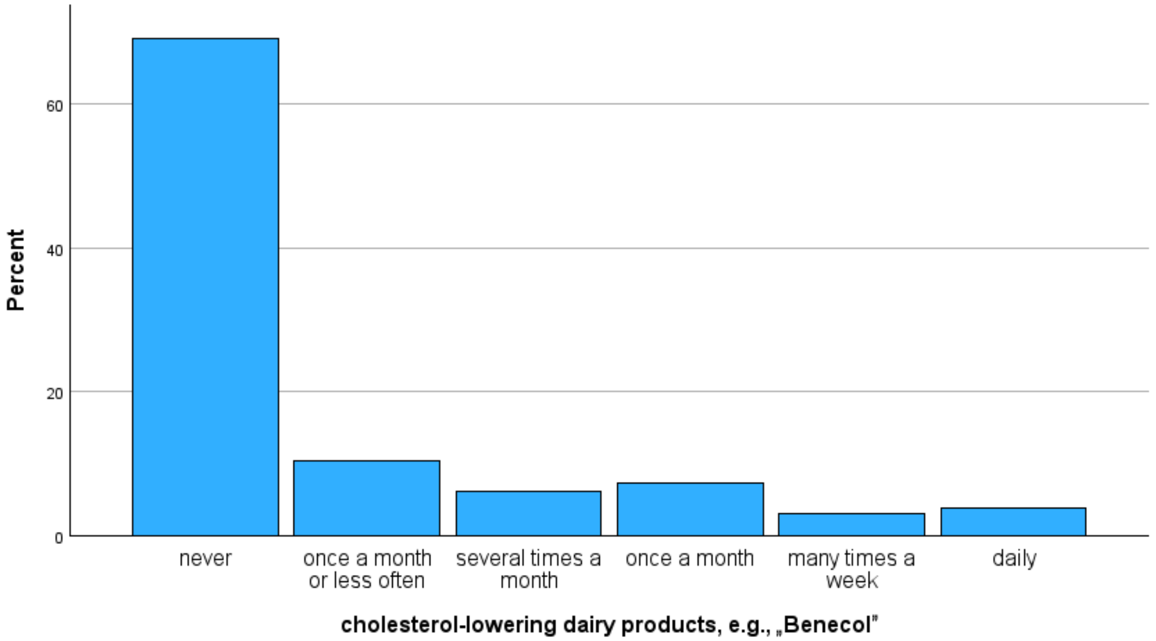

Figure S1M. Frequency of purchase of cholesterol-lowering dairy products

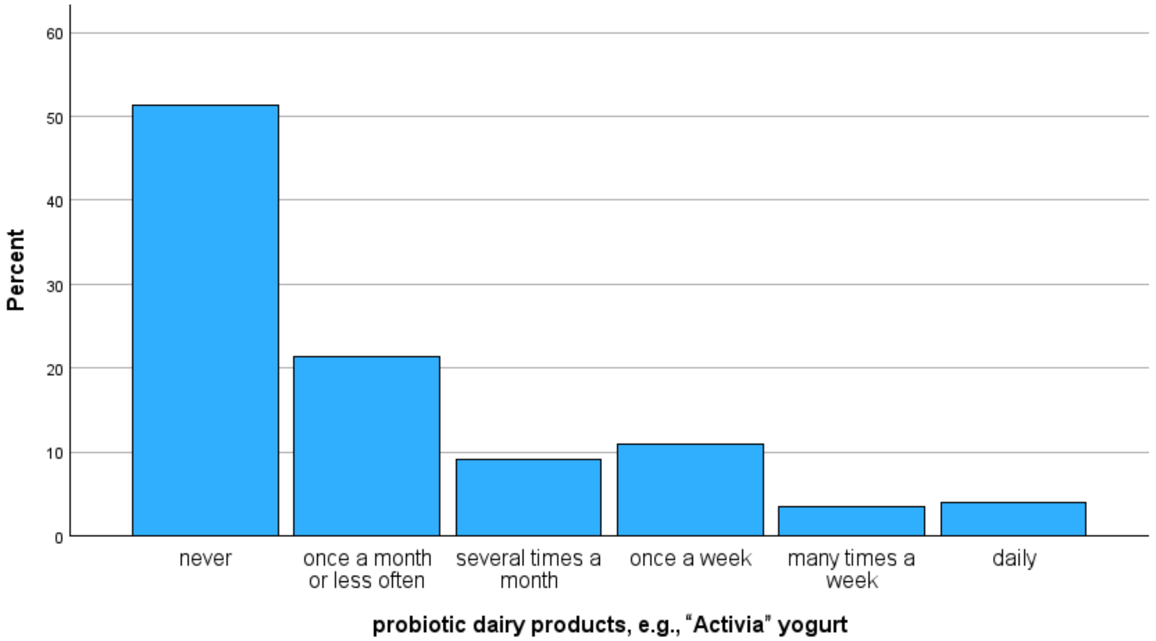

Figure S1N. Frequency of purchase of probiotic dairy products

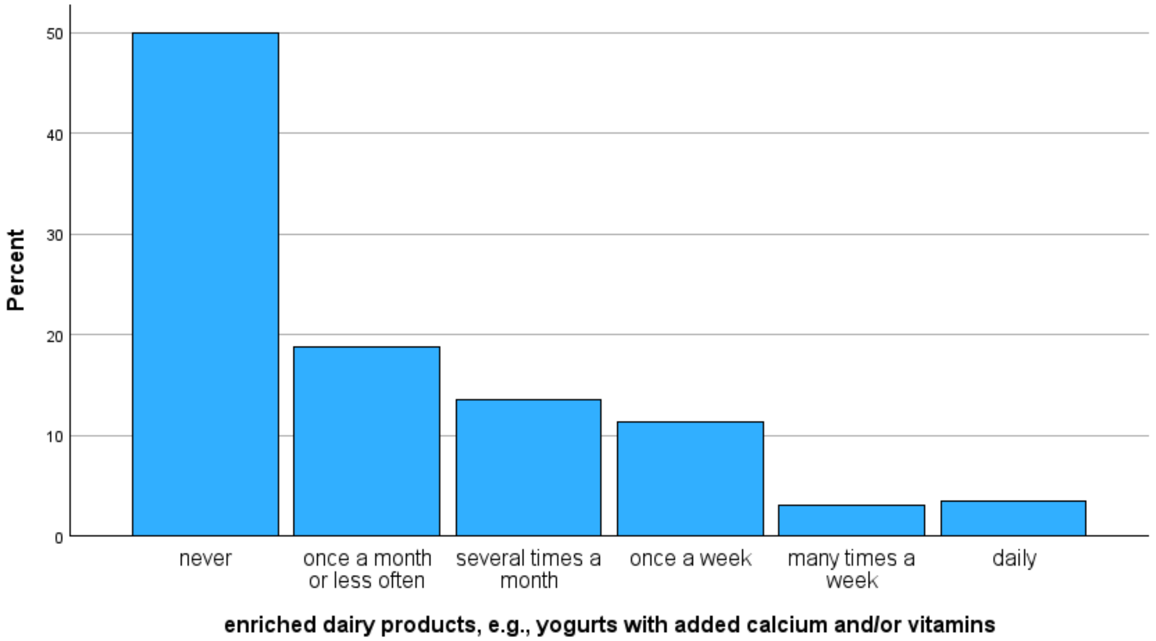

Figure S1O. Frequency of purchase of enriched dairy products

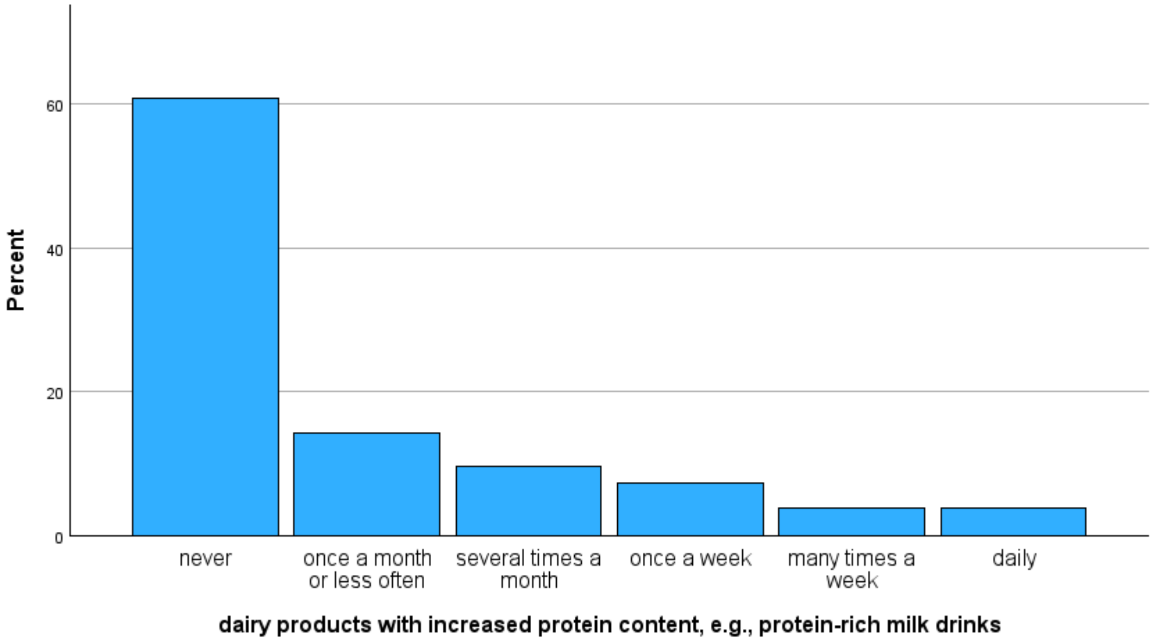

Figure S1P. Frequency of purchase of dairy products with increased protein content

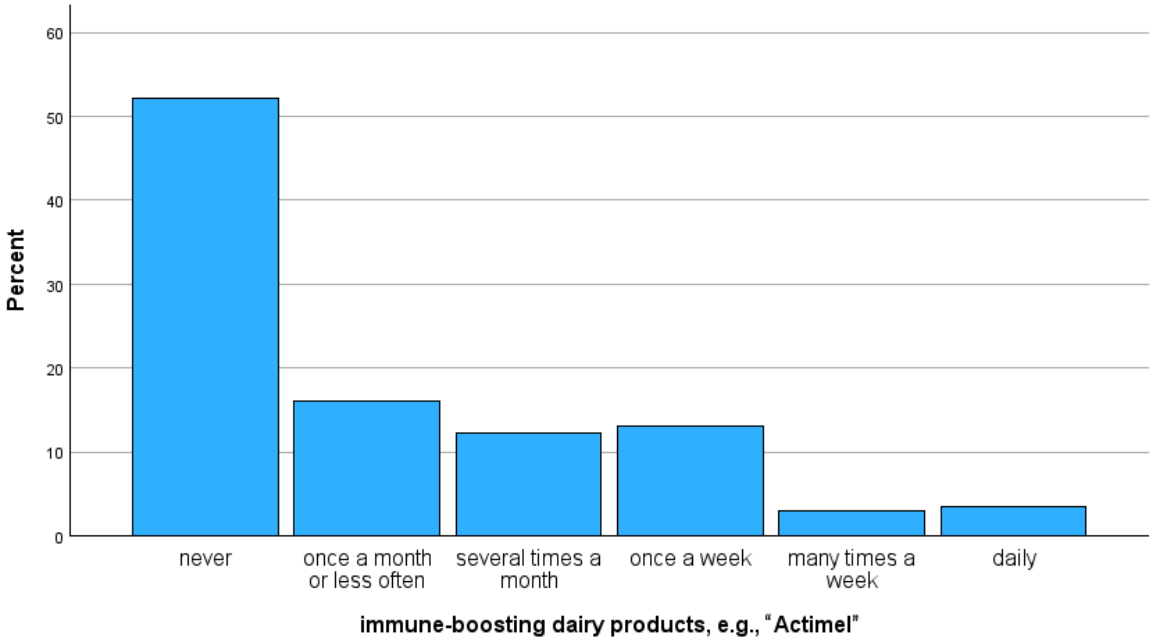

Figure S1R. Frequency of purchase of immune-boosting dairy products
